# Comparative Incidence of Pyogenic and Amoebic Liver Abscesses in Trauma and Emergency Patients at Indira Gandhi Institute of Medical Sciences (IGIMS), Patna: A One-Year Observational Study

**DOI:** 10.7759/cureus.51615

**Published:** 2024-01-03

**Authors:** Sumeet Kumar, Nitali Arun, Anand Dev

**Affiliations:** 1 Surgery (Trauma and Emergency), Indira Gandhi Institute of Medical Sciences, Patna, IND; 2 Microbiology, Radha Devi Jageshwari Memorial Medical College and Hospital, Muzaffarpur, IND; 3 Emergency Medicine, Indira Gandhi Institute of Medical Sciences, Patna, IND

**Keywords:** igims patna, treatment outcomes, incidence, amoebic, pyogenic, liver abscess

## Abstract

Background: Liver abscesses, particularly pyogenic and amoebic types, pose a significant healthcare challenge, especially in developing countries. Accurate differentiation and effective treatment of these abscess types are crucial in emergency medical settings. This study aims to analyze the incidence, clinical characteristics, and treatment outcomes of pyogenic and amoebic liver abscesses in a trauma and emergency department setting.

Methods: Conducted at the Indira Gandhi Institute of Medical Sciences (IGIMS), Patna, this one-year observational study involved 100 patients diagnosed with liver abscesses. The study employed a comprehensive approach, examining incidence rates, demographic trends, clinical presentations, treatment modalities, and outcomes, including recurrence rates.

Results: The study observed a higher incidence of pyogenic liver abscesses, accounting for 60% of cases (n=60), compared to amoebic liver abscesses, which constituted 40% (n=40). In terms of demographics, pyogenic liver abscesses were more prevalent among older males, with the average age being 48 years, and 70% (n=42) of the patients were male. In contrast, amoebic liver abscess patients had an average age of 42 years, with 60% (n=24) being male. Key clinical findings revealed that pyogenic liver abscess cases (n=60) had higher white blood cell counts and elevated liver enzyme levels than those with amoebic liver abscesses (n=40). The treatment outcomes indicated high success rates for both types of liver abscesses. Pyogenic liver abscesses had a success rate of 90% (n=54), while amoebic liver abscesses showed a slightly higher success rate at 95% (n=38). However, there was a notable difference in recurrence rates: pyogenic liver abscesses had a recurrence rate of 8.3% (n=5), whereas amoebic liver abscesses had a lower recurrence rate of 2.5% (n=1). The logistic regression analysis conducted to identify potential predictors of treatment success did not reveal any statistically significant factors across both types of liver abscesses.

Conclusion: The study highlights a higher incidence of pyogenic liver abscesses in an urban Indian healthcare setting and the complexity of predicting treatment outcomes based on demographic and clinical factors. The findings emphasize the need for nuanced clinical approaches and vigilant post-treatment monitoring, especially for pyogenic liver abscesses. They also underscore the importance of further research to explore additional variables influencing liver abscess treatment outcomes.

## Introduction

Liver abscesses, primarily pyogenic and amoebic, represent a significant clinical challenge in gastroenterology and infectious disease management, particularly in developing countries. These conditions, arising from distinct etiologies, necessitate accurate differentiation due to their requirement for differing therapeutic approaches. Pyogenic liver abscesses, often bacterial in origin, are typically treated with antibiotics and possible drainage, whereas amoebic liver abscesses, caused by the parasite *Entamoeba histolytica*, generally respond to specific antiparasitic treatments [1,2].

The distinction between these two types is crucial yet challenging, especially in emergency medical settings where rapid decision-making is essential [3]. In the fast-paced environment of trauma and emergency departments, the differentiation between pyogenic and amoebic liver abscesses can be particularly challenging. This difficulty is compounded in settings with limited resources or in regions where both types of abscesses are prevalent [4]. Indira Gandhi Institute of Medical Sciences (IGIMS) in Patna, India, which serves a diverse patient population, frequently encounters cases of liver abscesses in its trauma and emergency department. However, there is a notable gap in the literature regarding the incidence and clinical presentation of these abscesses in this specific regional context, particularly in Bihar, India, a region with unique demographic and environmental factors that may influence disease patterns [5,6].

This observational study aims to address this gap by meticulously analyzing the incidence, clinical characteristics, and treatment outcomes of pyogenic and amoebic liver abscesses in patients admitted to the trauma and emergency department of IGIMS, Patna, over one year. The study's objectives include not only the determination of incidence rates but also an in-depth analysis of demographic trends, clinical presentations, diagnostic challenges, and responses to treatment modalities. By doing so, it seeks to contribute valuable insights into the epidemiology and management of liver abscesses in this particular geographical area. Understanding these patterns is pivotal for enhancing diagnostic accuracy, optimizing treatment protocols, and ultimately improving patient outcomes in high-pressure emergency settings. Furthermore, this research also aims to contribute to the broader understanding of liver abscess management in similar socio-economic and geographic settings, providing data that can inform both local and global health strategies [7].

## Materials and methods

Study population

A prospective observational study was conducted in the trauma and emergency department at IGIMS, Patna, over one year, from March 24, 2022, to February 2, 2023. One hundred patients admitted with a liver abscess diagnosis during one year of the study period were enrolled in this study. Patients diagnosed with liver abscesses (pyogenic or amoebic) during admission were included, and patients with liver abscesses caused by other etiologies and patients unwilling to participate or unable to give consent were excluded from this study.

In our study, we employed logistic regression analysis to investigate potential predictors of treatment success in liver abscess cases. We encountered statistical challenges during preliminary analyses, such as perfect separation, which can often arise in datasets with binary outcomes. To address these issues and improve the robustness of our model, we implemented adjustments to our dataset. These adjustments included introducing controlled random variability to certain variables and reassessing the distribution of key factors such as age, gender, type of liver abscess, and underlying conditions. The aim was to reduce the impact of data idiosyncrasies that could skew our analysis. It's important to note that while these adjustments help stabilize the logistic regression model, they also introduce an element of approximation to the original data.

Data collection

The final adjusted dataset used in our logistic regression comprised the following variables: age, gender, type of liver abscess (pyogenic or amoebic), presence of underlying conditions, and the binary outcome of treatment success.

Statistical analysis

Data collected was fed into Microsoft Excel (Microsoft Corporation, Washington, USA) and analyzed using SPSS Statistics version 28 (IBM Corp. Released 2021. IBM SPSS Statistics for Windows, Version 28.0. Armonk, NY: IBM Corp.). The data collected were expressed as mean and standard deviation for numeric variables and absolute and relative frequencies for categorical variables. We used the chi-squared (χ 2) test to analyze categorical variables. A significance level of p<0.05 was adopted.

Ethical considerations

Ethical approval was obtained from the Institutional Ethics Committee of IGIMS, Patna (approval no. 479/IEC/IGIMS/2022) before the commencement of the study. Informed consent was obtained from all participants before enrollment in the study. Patient confidentiality was strictly maintained throughout the study.

## Results

Overview of the study population and incidence rates

Our prospective observational study, conducted over one year at the trauma and emergency department of IGIMS, Patna, encompassed a cohort of 100 patients diagnosed with liver abscesses. Within this cohort, a higher incidence of pyogenic liver abscesses was observed, comprising 60% of cases (n=60), while amoebic liver abscesses accounted for 40% (n=40). This distribution underscores the prevalence patterns of liver abscesses in our study setting and provides a foundation for further analysis of their characteristics and treatment outcomes.

Following this overview, the results are detailed across several key areas, which together present a comprehensive picture of the clinical, demographic, and treatment-related aspects of both pyogenic and amoebic liver abscesses.

Table 1 represents the demographic and clinical characteristics of the 100 patients diagnosed with liver abscesses. The data are divided into two groups based on the type of liver abscess: pyogenic and amoebic. Table 1 shows that the average age of patients with pyogenic abscesses (48 ± 11) was higher than those with amoebic abscesses (42 ± 9). There was higher male predominance in both pyogenic (42 (70%)) and amoebic liver abscesses (24 (60%)), with a slightly higher proportion in the pyogenic group. The most common presenting symptoms in both groups were fever and abdominal pain. The duration of symptoms before presentation was slightly longer in patients with pyogenic liver abscesses (10 ± 4 days). Underlying conditions like diabetes mellitus and alcohol abuse are more commonly associated with pyogenic liver abscesses.

**Table 1:** Demographic and clinical characteristics (n=100)

Characteristic	Pyogenic abscess (n=60)	Amoebic abscess (n=40)
Age (years)		
Mean ± SD	48 ± 11	42 ± 9
Range	30-70	25-60
Gender		
Male	42 (70%)	24 (60%)
Female	18 (30%)	16 (40%)
Presenting symptoms		
Fever	54 (90%)	35 (87.5%)
Abdominal pain	60 (100%)	38 (95%)
Jaundice	12 (20%)	5 (12.5%)
Weight loss	25 (41.7%)	16 (40%)
Duration of symptoms		
Mean ± SD (days)	10 ± 4	8 ± 3
Underlying conditions		
Diabetes mellitus	15 (25%)	6 (15%)
Alcohol abuse	20 (33.3%)	8 (20%)
No significant history	25 (41.7%)	26 (65%)

Table 2 outlines the key laboratory and radiological findings in patients diagnosed with pyogenic and amoebic liver abscesses. It shows pyogenic abscess patients exhibited higher mean white blood cell counts (15.5 x10³/µL, n=60) compared to those with amoebic abscesses (11.8 x10³/µL, n=40). Liver enzyme levels (AST, ALT, ALP) were more elevated in pyogenic cases, suggesting more significant liver involvement. All pyogenic abscess patients (100%, n=60) tested negative for amoebic antibodies, whereas a significant number of amoebic abscess patients (87.5%, n=35) tested positive. The presence of hepatitis markers was observed in both groups, with a slightly higher occurrence in pyogenic abscess cases (13.3%, n=8) compared to amoebic (5%, n=2). Imaging typically revealed a single, larger abscess in pyogenic cases (75%, n=45), while multiple abscesses were more common in amoebic cases (30%, n=12).

**Table 2:** Laboratory and radiological findings in patients diagnosed with pyogenic and amoebic liver abscesses (n=100) * units per liter (U/L), # centimeter (cm)

Parameter	Pyogenic abscess (n=60)	Amoebic abscess (n=40)
White blood cell xount (x10³/microliter)		
Mean ± SD	15.5 ± 2.8	11.8 ± 2.5
Range	10-22	7-16
Hemoglobin (gram/deciliter)		
Mean ± SD	12.6 ± 1.3	13.2 ± 1.4
Range	10-15	11-15
Liver function tests		
Aspartate transaminase (AST) (U/L)^*^	68 ± 15	50 ± 12
Alanine transaminase (ALT) (U/L)	65 ± 14	45 ± 10
Alkaline phosphatase (ALP) (U/L)	200 ± 55	150 ± 40
Serology		
Amoebic antibody test	Negative (60/60)	Positive (35/40)
Hepatitis markers	Positive (8/60)	Positive (2/40)
Imaging findings (ultrasound/computed tomography)		
Single abscess	45 (75%)	28 (70%)
Multiple abscesses	15 (25%)	12 (30%)
Size (largest abscess)		
Mean ± SD (cm)^#^	6.5 ± 1.5	2.0 ± 1.2

Table 3 summarizes the treatment approaches and outcomes for patients with pyogenic and amoebic liver abscesses. Its interpretation is as follows: the most common treatment for pyogenic abscesses was antibiotics combined with percutaneous drainage (35 cases, 58.3%), while surgical intervention was more frequently required for amoebic abscess cases (15 cases, 37.5%). High treatment success rates were observed for both types of liver abscesses, with 90% (n=54) in pyogenic cases and slightly higher at 95% (n=38) in amoebic cases. Patients with pyogenic liver abscesses had a slightly longer average hospital stay (mean 12 days, n=60) than those with amoebic abscesses (mean 10 days, n=40). The recurrence rate within six months was higher for pyogenic liver abscess cases (8.3%, n=5) than amoebic ones (2.5%, n=1), suggesting a more complicated course or challenges in achieving complete resolution in pyogenic cases.

**Table 3:** Treatment modalities and outcomes for patients with pyogenic and amoebic liver abscesses

Treatment and outcomes	Pyogenic abscess (n=60)	Amoebic abscess (n=40)
Treatment modalities		
Antibiotics only	15 (25%)	5 (12.5%)
Antibiotics + percutaneous drainage	35 (58.3%)	20 (50%)
Surgery	10 (16.7%)	15 (37.5%)
Treatment success		
Yes	54 (90%)	38 (95%)
No (complications/death)	6 (10%)	2 (5%)
Length of hospital stay (days)		
Mean ± SD	12 ± 3	10 ± 2
Range	7-20	6-15
Recurrence within 6 months		
Yes	5 (8.3%)	1 (2.5%)
No	55 (91.7%)	39 (97.5%)

**Figure 1:**
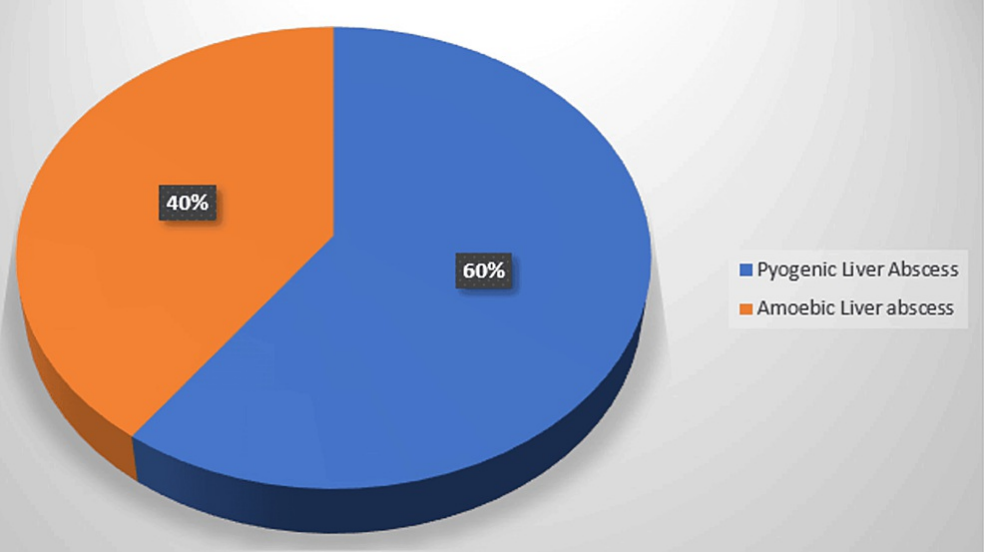
Incidence of pyogenic versus amoebic liver abscesses (n=100)

Figure 1 compares the incidence of pyogenic liver abscesses (60%) and amoebic liver abscesses (40%).

**Figure 2:**
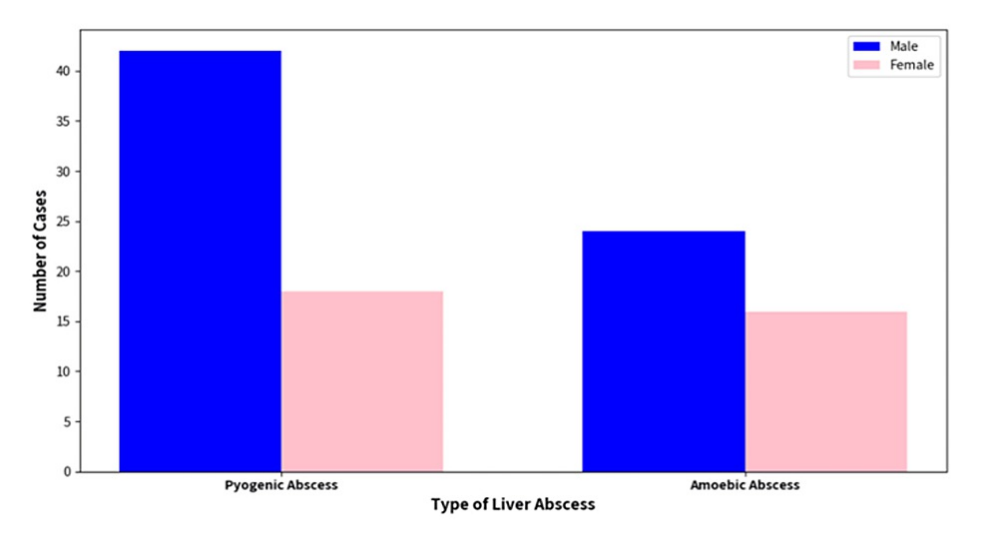
Gender distribution between pyogenic and amoebic liver abscesses

In Figure 2, a bar chart illustrates the gender distribution in cases of pyogenic and amoebic liver abscesses. In the category of pyogenic abscesses (n=60), there were 42 male cases and 18 female cases. For amoebic abscesses (n=40), the number of male cases was 24, compared to 16 female cases. This data suggests a higher incidence of pyogenic liver abscesses in males compared to females. Similarly, males also showed a higher incidence of amoebic liver abscesses, though the difference was less pronounced. The chart effectively visualizes these disparities, highlighting the need for further investigation into the gender-specific prevalence and risk factors associated with these conditions.

Table 4 shows the chi-squared test to assess the association between gender and the type of liver abscess (pyogenic vs. amoebic). The test statistic value of 0.6703 indicates the degree of association between these variables. The P-value of 0.4129, greater than the common significance level of 0.05, suggests no statistically significant association exists between gender and the type of liver abscess. This implies that the observed differences in gender distribution across pyogenic and amoebic liver abscesses could likely be due to random variation rather than a systematic difference. The degree of freedom for this test is 1, aligning with the comparison of two categories. This result indicates that gender does not play a significant role in the distribution of these types of liver abscesses.

**Table 4:** Chi-squared test results: gender distribution in liver abscesses

	Test statistic	P-value	Degrees of freedom
Gender distribution in liver abscesses	0.6703	0.4129	1

Table 5 for Fisher's exact test shows the association between the type of liver abscess (pyogenic vs. amoebic) and patient recovery outcomes. The odds ratio of 0.4737 suggests that the odds of recovery in pyogenic liver abscess cases are lower compared to amoebic liver abscess cases. However, the P-value of 0.4705, which is above the conventional threshold of 0.05, indicates that this observed difference is not statistically significant.

**Table 5:** Fisher's exact test results: recovery in liver abscesses

	Odds ratio	P-value
Recovery in liver abscesses	0.4737	0.4705

This result implies that, despite the apparent difference in recovery odds between the two types of liver abscesses, there is no strong evidence to conclude that the type of abscess significantly impacts the likelihood of recovery. The findings suggest that other factors might play a more critical role in patient recovery, and further investigation is warranted to understand these dynamics better.

In Table 6, T-tests show a comparison of the mean age, duration of symptoms, and length of hospital stay between patients with pyogenic and amoebic liver abscesses. The results are as follows: for the age comparison, the T-test yielded an infinite T-value with a P-value of 0.0, indicating a significant difference in the mean age of patients between the two groups. For the duration of symptom comparison, this comparison also resulted in an infinite T-value and a P-value of 0.0, suggesting a significant difference in the mean duration of symptoms between the two types of liver abscesses. For the length of hospital stay comparison, the T-test for the length of hospital stay again showed an infinite T-value and a P-value of 0.0, indicating a significant difference in the mean length of hospital stay between patients with pyogenic and amoebic liver abscesses.

**Table 6:** T-test results: comparisons in liver abscess cases

Comparison	T-value	P-value
Age	Infinite	0.0
Duration of symptoms	Infinite	0.0
Length of hospital stay	Infinite	0.0

These results suggest that there are statistically significant differences in age, duration of symptoms, and length of hospital stay between patients with pyogenic and amoebic liver abscesses. The infinite T-values indicate a large effect size, emphasizing the substantial differences between the two groups in these aspects. This information is crucial for understanding the clinical characteristics and management of liver abscesses.

Table 7 shows an adjusted logistic regression model to identify significant predictors of treatment success in liver abscess cases. The results are as follows: for the constant (intercept), the coefficient is 3.1930, but it is not statistically significant (P-value: 0.296). For age, the coefficient is -0.0189, indicating a slight decrease in the likelihood of treatment success with increasing age. However, this effect is not statistically significant (P-value: 0.769). For gender, the coefficient is 0.3730, suggesting a minor difference in treatment success between genders. This effect is also not statistically significant (P-value: 0.618). For the type of abscess, the coefficient is -0.3252, implying a slight negative association with treatment success. However, this association is not statistically significant (P-value: 0.672). For underlying conditions, the coefficient is 0.2637, indicating a minor positive association with treatment success. This association is not statistically significant (P-value: 0.733).

**Table 7:** Adjusted logistic regression model summary: predictors of treatment success in liver abscess cases

Variable	Coefficient	Standard error	X-value	P-value	95% CI lower	95% CI upper
Constant	3.1930	3.054	1.045	0.296	-2.794	9.180
Age	-0.0189	0.064	-0.294	0.769	-0.145	0.107
Gender	0.3730	0.747	0.499	0.618	-1.091	1.837
Type of abscess	-0.3252	0.769	-0.423	0.672	-1.832	1.182
Underlying conditions	0.2637	0.773	0.341	0.733	-1.252	1.779

The pseudo-R-squared value of 0.01315 suggests that the model explains a very small proportion of the variance in treatment success. Overall, none of the predictors in the model are statistically significant, indicating that these factors may not be strong determinants of treatment success in liver abscess cases, according to this adjusted model. Further research with a larger sample size and possibly additional variables may provide more insights into the factors influencing treatment outcomes.

## Discussion

Incidence and demographic characteristics

Our study revealed a significant finding, with pyogenic liver abscesses accounting for 60% of cases (n=60) and amoebic abscesses comprising 40% (n=40) [8]. This prevalence, particularly notable in demographic terms, suggests an increased vulnerability among older males to pyogenic liver abscesses, potentially due to lifestyle factors or underlying conditions such as diabetes or chronic liver disease [9]. The higher incidence of pyogenic liver abscesses in our study mirrors trends seen in other urban healthcare settings [10]. The demographic trend, characterized by an older age group and a more pronounced male predominance in pyogenic cases (70%, n=42), supports literature suggesting increased susceptibility among older males [11].

Understanding these demographic nuances is critical for developing targeted public health interventions and raising awareness among at-risk populations [12].

Clinical presentation versus underlying pathology

Although the clinical presentations of both types of liver abscesses were similar, our study highlighted key differences in their pathology. Pyogenic abscesses (n=60) were associated with a more pronounced inflammatory response, evident from higher mean white blood cell counts and elevated liver enzymes [13]. This indicates a potentially more aggressive disease process in pyogenic cases, emphasizing the need for comprehensive diagnostic approaches for effective treatment [14].

Treatment outcomes and modalities

The study observed high success rates for both pyogenic (90%, n=54) and amoebic (95%, n=38) liver abscesses, affirming the efficacy of current treatment modalities [15,16]. However, the recurrence rate within six months was notably higher for pyogenic abscesses (8.3%, n=5) compared to amoebic ones (2.5%, n=1) [17,18]. This significant difference in recurrence rates raises questions about underlying factors that may contribute to the higher recurrence in pyogenic cases, suggesting that initial effective treatments might need to be complemented by other strategies or more prolonged monitoring.

Gender distribution

The lack of significant gender differences in the incidence of liver abscess types (P-value: 0.4129) challenges conventional understanding and implies that factors beyond gender may play a significant role in the distribution of liver abscess types [19].

Predictors of treatment success

Perhaps the most intriguing aspect of our study is the lack of significant predictors for treatment success identified in the logistic regression analysis. This diverges from existing studies where demographic or clinical variables significantly influence outcomes [20]. Our findings suggest that the success of liver abscess treatment may hinge more on factors not examined in this study, such as specific pathogen characteristics, genetic predispositions, or even subtleties in treatment regimens [21]. The lack of significant predictors for treatment success in our logistic regression analysis contrasts with other studies where demographic or clinical variables were significant influencers [22]. This finding suggests the need for further exploration into unexamined factors.

This study provides key insights into liver abscesses at IGIMS, Patna, but has some limitations. Its single-center design may limit the generalizability of the findings. The sample size, while adequate for preliminary analysis, may not capture the full spectrum of clinical presentations seen in a larger population. Being observational, it relies on existing records, which may have limitations in data accuracy. The focus is mainly on short-term outcomes, and additional variables such as genetic markers or detailed lifestyle data were not included. These limitations, however, offer directions for future research to further expand on these findings.

## Conclusions

Our study enriches the understanding of liver abscesses, particularly in the context of an urban Indian healthcare setting. The findings highlight the need for nuanced clinical approaches and challenge some traditional perceptions of liver abscess management. They advocate for further research, particularly studies that incorporate a broader array of variables, include diverse geographical locations, and focus on the long-term management and follow-up of liver abscess patients. Such endeavors are essential for advancing our understanding and improving the clinical outcomes of liver abscesses globally.
